# Off-target binding of the histone deacetylase inhibitor vorinostat to carbonic anhydrase II and IX

**DOI:** 10.1107/S2053230X25007447

**Published:** 2025-08-26

**Authors:** Mitchell C. Gulkis, James T. Hodgkinson, Céleste P. Sele, Wolfgang Knecht, Robert McKenna, S. Zoë Fisher

**Affiliations:** a Department of Biochemistry and Molecular Biology University of Florida 1149 Newell Drive Gainesville FL32610 USA; b Leicester Institute of Structural and Chemical Biology and School of Chemistry University of Leicester University Road LeicesterLE1 7RH United Kingdom; c Lund Protein Production Platform and Protein Production Sweden, Department of Biology Lund University Sölvegatan 35 22362Lund Sweden; d Science for Life Laboratory, Department of Biology Lund University Sölvegatan 35 22362Lund Sweden; e European Spallation Source ERIC PO Box 176 22100Lund Sweden; Stanford Synchrotron Radiation Lightsource, USA

**Keywords:** HDAC inhibitors, SAHA, vorinostat, human carbonic anhydrases, off-target binding

## Abstract

Histone deacetylase inhibitors (HDACi) are widely used in cancer therapy but often suffer from off-target effects due to their pan-inhibitory activity towards zinc-dependent enzymes. Vorinostat (SAHA), a hydroxamate-based HDACi, has been shown to lack isoform selectivity, potentially leading to unintended interactions with other metalloenzymes. Here, we report high-resolution crystal structures of SAHA bound to human carbonic anhydrase II (CA II) and a carbonic anhydrase IX (CA IX) active-site mimic. Structures determined at room temperature and 100 K revealed two distinct SAHA conformers in both CA II and the CA IX mimic, with the hydroxamate moiety displacing the zinc-bound water and adopting either a tetrahedral or pentahedral coordination to Zn^2+^. Differences in hydrophobic interactions were observed between CA II and the CA IX mimic due to the F131V amino-acid difference between the two enzymes. *SwissDock* modeling accurately predicted the SAHA binding orientations observed in crystallography. Thermal shift assays using nanoDSF showed minimal stabilization of either CA by SAHA, in contrast to the potent CA inhibitor acetazolamide. Binding-energy calculations suggest that SAHA may bind carbonic anhydrases with affinities comparable to its HDAC targets. These findings highlight potential off-target binding of SAHA to carbonic anhydrases, which may contribute to its clinical side effects. The results also suggest that hydroxamates may serve as a nonsulfonamide scaffold for novel CA inhibitors, although isoform selectivity remains a challenge.

## Abstract 2

We determined high-resolution crystal structures of the histone deacetylase inhibitor vorinostat bound to both human carbonic anhydrase II and a carbonic anhydrase IX active-site mimic. Thermal shift assays using differential scanning fluorimetry showed minimal stabilization of either carbonic anhydrase by vorinostat, in contrast to the potent carbonic anhydrase inhibitor acetazolamide.

## 1. Introduction

**Figure 1:**
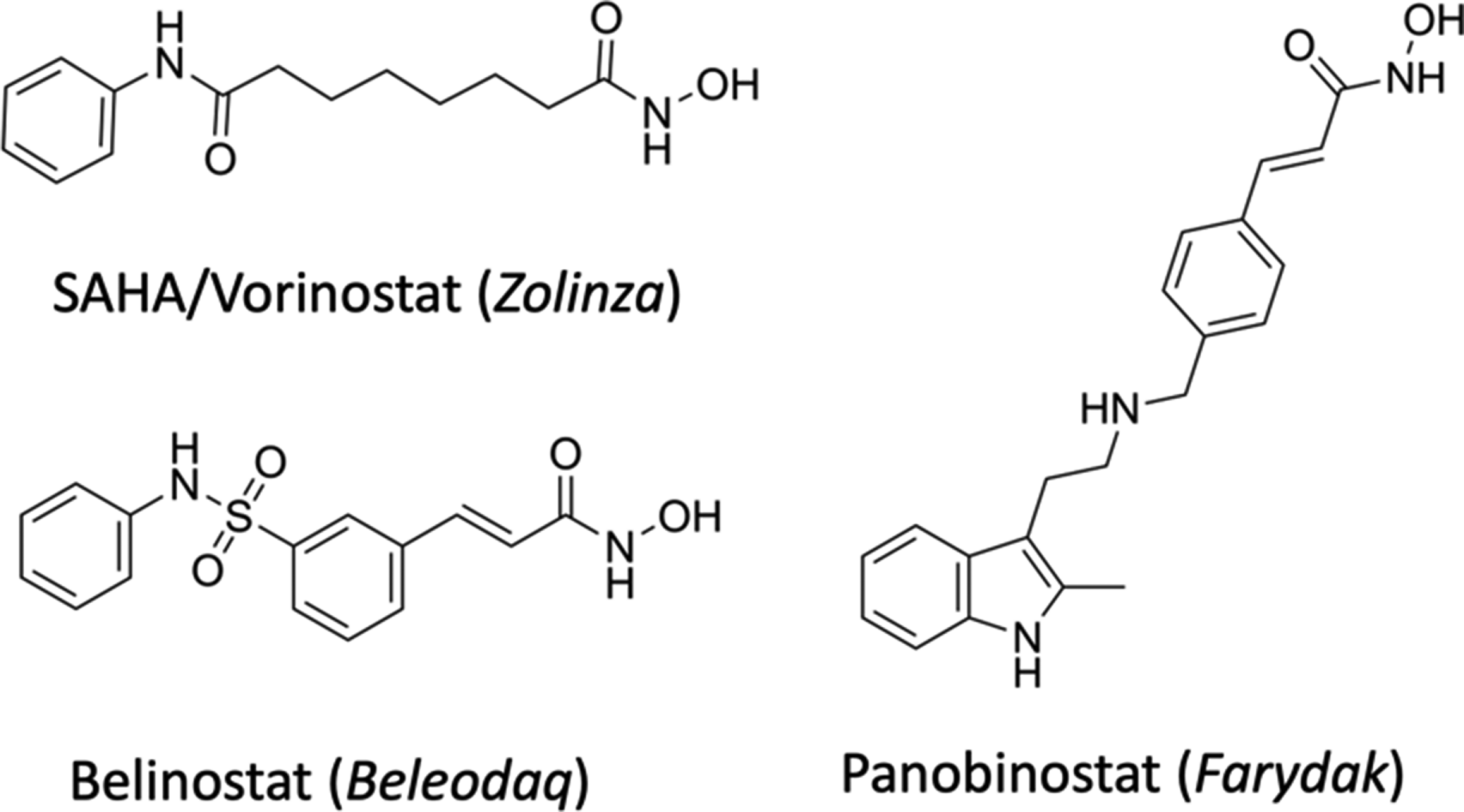
Clinically used HDAC inhibitors based on a hydroxamate zinc-binding group. The commercial names are indicated in parentheses.

Over the past three decades, histone deacetylase inhibitors (HDACi) have emerged as promising therapeutic agents in the treatment of cancers, with six drugs currently in clinical use (Han *et al.*, 2023 ▸; Ho *et al.*, 2020 ▸). Histone deacetylases catalyze the hydrolysis of acetyl functional groups of *N*-acetylated lysine residues in histone and nonhistone proteins. With regard to histone proteins, this modification plays an important role in chromatin structure and gene transcription. HDAC inhibitors perturb the expression of genes involved in cell-cycle regulation, DNA damage repair and apoptosis pathways. HDACs can be categorized into class I (HDAC1, HDAC2, HDAC3 and HDAC8), class IIa (HDAC4, HDAC5, HDAC7 and HDAC9), class IIb (HDAC6 and HDAC10), class III (SIRT1-SIRT7) and class IV (HDAC11). All of the HDACs, aside from class III, contain a divalent zinc ion in their catalytic active site. Vorinostat (suberoylanilide hydroxamic acid or SAHA) was the first HDACi to obtain FDA approval, in 2006, for the treatment of cutaneous T-cell lymphoma. This was followed by other drugs, including belinostat and panobinostat (Fig. 1 ▸; Ho *et al.*, 2020 ▸; Falkenberg & Johnstone, 2014 ▸). Studies have shown that SAHA is a pan-HDACi for all 11 zinc-dependent HDACs, with IC_50_ values in the low- to high-nanomolar range. However, SAHA binds to several off-target proteins, reducing the specificity of this drug (Ho *et al.*, 2020 ▸; Lechner *et al.*, 2022 ▸). The pan-zinc-dependent HDAC inhibition of these drugs, combined with associated off-targets, has been hypothesized to be responsible for their side effects in patients (Ho *et al.*, 2020 ▸; Lechner *et al.*, 2022 ▸) and their limited applicability to hematological cancers due to dose-limiting toxicities in treating patients with solid tumors (Shah, 2019 ▸; Bradley *et al.*, 2009 ▸; Vansteenkiste *et al.*, 2008 ▸). As such, there have been concerted efforts to modify existing HDACi to introduce HDAC isoform specificity to improve the targeting of particular diseases and to reduce the severe side effects associated with current HDACi used in the clinic (Steimbach *et al.*, 2022 ▸; Liu *et al.*, 2020 ▸; Patel *et al.*, 2023 ▸).

SAHA is comprised of three chemical moieties: the hydroxamate zinc-binding group, a hydrophobic hexyl linker and a terminal phenylamide (anilide) group (Fig. 1 ▸). The zinc coordination of the hydroxamate moiety was first observed in the crystal structure of HDAC8 in complex with SAHA (Somoza *et al.*, 2004 ▸; PDB entry 1t69). Since then, there have been several structures of SAHA bound to other class I HDACs and, interestingly, also a class II HDAC-like protein from bacteria (Nielsen *et al.*, 2005 ▸; Lauffer *et al.*, 2013 ▸).

Another family of zinc metalloenzymes are the α-class carbonic anhydrases (CAs), which catalyze the reversible hydration of carbon dioxide to produce bicarbonate and a proton, H^+^ (Silverman, 1995 ▸). The first class of CA inhibitors (CAi) to be discovered, such as acetazolamide (AZM), were sulfonamide-based compounds developed in the 1950s to treat congestive heart failure and later glaucoma. The inhibitory mechanism of the sulfonamide drugs is by direct binding to the zinc ion and displacing the catalytic zinc-bound water. In recent decades, research into CA isoform IX has shown it to be overexpressed in over 30 solid, hypoxic tumors (Chegwidden, 2021 ▸). Currently, a small-molecule carbonic anhydrase inhibitor (CAi), SLC-0111, for the treatment of hypoxic tumors is in Phase Ib/II clinical trials. Drug design to target CA IX specifically is complicated, just like for HDACi, by the very high active-site conservation among other CA isoforms: >80% in most cases (Supuran, 2020 ▸). As such, there is significant interest in detailed studies of drug-binding interactions and medicinal chemistry approaches to create CA isoform-selective drugs that will have reduced off-target binding and side effects.

While supporting very different enzyme-mediated reactions, the α-class CAs and HDAC class I enzymes share a similar active site, with both containing Zn^2+^ at the bottom of a conical active site lined with both hydrophilic and hydrophobic amino-acid residues. Since full-length CA IX is a multi-domain, dimeric membrane protein, an engineered form of CA II was used as a template to approximate or ‘mimic’ the CA IX active site. Briefly, the CA IX mimic construct was developed by Pinard and coworkers by substituting seven residues in and around the active site of wild-type (WT) CA II to create a proxy for the native CA IX catalytic domain for crystallographic studies (Supplementary Fig. S1). In this way, the attractive practical properties of WT CA II, *i.e.* solubility, ease of expression and robust crystallization, are retained while providing a working analog of the CA IX active site (Genis *et al.*, 2009 ▸; Pinard *et al.*, 2015 ▸).

In this study, we describe the *SwissDOCK* predicted binding of SAHA to CA II and CA IX and experimental verification by X-ray crystallography. We report room-temperature crystal structures of both CA II and the CA IX mimic in complex with the HDACi SAHA and a cryogenic crystal structure of the CA IX mimic with SAHA. The structures reveal different binding modes for SAHA in the different CA constructs. Furthermore, the hydroxamate zinc-binding group of SAHA occupies two different zinc-coordination positions within each structure. In addition to crystal structures, we also determined the relative thermal destabilization of CA II and CA IX by SAHA and the known CAi AZM using nano differential scanning fluorimetry (nanoDSF).

## 2. Materials and methods

### 2.1. Protein production of WT CA II and the CA IX mimic

The cDNA encoding WT CA II was cloned into a pET vector under ampicillin selection (Tanhauser *et al.*, 1992 ▸). The relevant sequences and mutations to adapt the CA II active site into the CA IX mimic are shown in Supplementary Fig. S1. Briefly, the seven mutations relative to WT are as follows: A65S, N67Q, E69T, I91L, F131V, K170E and L204A (Genis *et al.*, 2009 ▸; Pinard *et al.*, 2015 ▸). The cDNA sequence encoding the human CA IX mimic was codon-optimized for expression in *Escherichia coli*, synthesized and cloned into pET-26b(+) (Novagen) under kanamycin selection by GenScript (Hong Kong). Both plasmids were transformed into chemically competent *E. coli* Tuner(DE3) cells (Novagen).

Both CA constructs were expressed in LB medium. Overnight starter cultures were prepared by inoculating 50 ml fresh LB medium from a frozen glycerol stock of *E. coli* Tuner (DE3) cells transformed with the respective plasmid. The preculture was grown overnight at 200 rev min^−1^ at 37°C (310 K) with either 100 or 50 µg ml^−1^ ampicillin or kanamycin for CA II and the CA IX mimic, respectively. The next day, 2 × 2 l fresh LB medium, supplemented with appropriate antibiotics, was inoculated by adding 10 ml overnight culture to every litre of LB. The cultures were grown in TunAir baffled flasks at 225 rev min^−1^ at 37°C (310 K). When the OD_600_ reached 1.0–1.2, protein expression was induced by the addition of 1 m*M* (final concentration) isopropyl β-d-1-thio­galactopyranoside (IPTG) and the medium was supplemented with 1 m*M* ZnSO_4_. Expression continued overnight at 20°C (293 K). After approximately 18 h, the cells were harvested by centrifugation in a JLA-8.1 rotor (Beckman) at 6000*g* for 20 min at 4°C (277 K). The cell pellets were frozen at −80°C (193 K) until further processing.

The frozen cell pellets were thawed and resuspended by dissolving them in 50 ml wash buffer 1 (0.2 *M* sodium sulfate, 0.1 *M* Tris–HCl pH 9.0) for every cell pellet from a 1 l culture. The cells were lysed by the addition of 20 mg lysozyme (Merck) and 1 mg DNase I (Merck) per cell pellet from a 1 l culture. This mixture was stirred overnight in the cold room. After approximately 18 h, the cell lysates were clarified by centrifugation in a JA-25.50 rotor (Beckman) at ∼20 000*g* for 90 min at 4°C (277 K).

The protein was purified at ambient temperature using the CA-specific affinity-chromatography medium *p*-aminomethylbenzenesulfonamide–agarose resin (pAMBS; Sigma–Aldrich) pre-packed in a disposable gravity column. The resin was pre-equilibrated with wash buffer 1. After loading the clarified lysate, the columns were washed with ∼20 column volumes (CV) of wash buffer 1, followed by ∼20 CV of wash buffer 2 (0.2 *M* sodium sulfate, 0.1 *M* Tris–HCl pH 7.0). The UV–Vis signal of the wash fraction was monitored at 280 nm until all nonspecifically bound proteins and DNA were washed off. The proteins were eluted from the column with 10 CV of elution buffer (0.4 *M* sodium azide, 50 m*M* Tris–HCl pH 8.0). The elution fraction was filtered through 100 kDa molecular-weight cutoff (MWCO) Amicon Ultra filtration units to remove high-molecular-weight proteins that co-purified. The filtrate from this step was then concentrated and buffer-exchanged into 50 m*M* Tris–HCl pH 8.0 using Swissci reverse concentrators with 10 kDa MWCO. The protein purity was confirmed using SDS–PAGE and the final concentration was determined to be 14–15 mg ml^−1^ by measuring the UV absorbance at 280 nm. Typical yields for both purified CAII and CA IX mimic proteins are around 20–50 mg per litre of cell culture.

### 2.2. Protein crystallization and complex formation

Apo crystals were prepared of both WT CA II (14 mg ml^−1^) and the CA IX mimic (15 mg ml^−1^) using two different crystallization conditions: 2.8 *M* ammonium sulfate, 0.1 *M* Tris–HCl pH 8.0 or 1.25 *M* sodium citrate, 0.1 *M* Tris–HCl pH 8.0. Multiple drops were prepared in varying volumes (5, 10 and 20 µl) using 1:1 protein-to-precipitant ratios in sitting-drop 24-well Linbro plates with 1 ml precipitant solution in the reservoir. Crystal plates were incubated at 20°C (293 K) and typically appeared overnight (using the ammonium sulfate condition) or within a week (using the sodium citrate condition). The CA crystals used in this study are usually plate-shaped with sizes in the range 200–500 × 40–100 µm.

The HDAC inhibitor suberoylanilide hydroxamic acid (SAHA or vorinostat; Merck) was dissolved in 100% DMSO to a concentration of 100 m*M*. Three different approaches were taken to prepare the complex: (i) soaking, (ii) co-crystallization and (iii) transferring crystals onto dried compound.

Drug soaks were set up by adding dissolved SAHA to drops containing crystals in a 1:10 protein:SAHA molar ratio. However, the presence of 10% DMSO was very damaging and the crystals were destroyed within 1 h. Furthermore, it seems that the ammonium sulfate condition caused the drug to immediately come out of solution, while it appeared that more of the SAHA stayed in solution with sodium citrate. Subsequent diffraction experiments were performed with crystals grown in the citrate condition only.

Co-crystals were prepared by mixing the protein and drug solution in a 1:10 ratio and incubating at room temperature for 1 h. Since the drug is quite insoluble, the mixture was centrifuged at 13 000*g* and crystallization drops were prepared with the supernatant. Besides soaking and co-crystallization, crystals were also transferred onto dried compound in sitting-drop microbridges. Briefly, 2 µl 100 m*M* SAHA in 100% DMSO was pipetted onto microbridges in 24-well Linbro plates. These were left open in a fume hood overnight to evaporate off the DMSO. The next day, the mother liquor from apo CA crystal plates were transferred onto the dried compound and crushed with the pipette tip. Next, crystals were moved into these drops and left for over a week prior to testing with X-rays.

### 2.3. Modeling drug binding using *SwissDock*

To assess the feasibility of preparing SAHA–CA complexes, the web-based docking program *Docking with Attracting Cavities*, as implemented in *SwissDock*, was used to model and estimate possible interactions (Röhrig *et al.*, 2023 ▸; Bugnon *et al.*, 2024 ▸). The predicted binding modes of SAHA in the active-site cavity of CA II and the CA IX mimic were calculated after removing water molecules but leaving the zinc ion in place. A search box of 7 × 8 × 7 Å was defined around the active site and five random initial conditions was selected. The output was inspected using *ChimeraX* 1.8 (Meng *et al.*, 2023 ▸) and *PyMOL* (v. 2.5.2; Schrödinger).

### 2.4. Room-temperature and cryo X-ray data collection

Three crystals (WT-transfer, WT-co-crystal and Mimic-transfer) were prepared for X-ray diffraction data collection by scooping crystals with 500 µm cryoloops (MiTeGen). For room-temperature measurements, the crystals were protected from dehydration by covering the loop with 3–4 cm long quartz capillaries (Vitrocom, New Jersey, USA) filled at the opposite end with 10–15 µl precipitant solution and sealed with beeswax. For data collection at 100 K, the crystals were quick-dipped into 10 µl reservoir solution supplemented with 20% glycerol prior to flash-cooling in liquid nitrogen. All diffraction data were collected on the BioMAX beamline at MAX IV Laboratory, Lund, Sweden (Ursby *et al.*, 2020 ▸). The data sets were processed on-site using the automated pipelines as implemented at BioMAX. Outputs from the *autoPROC* pipeline (Vonrhein *et al.*, 2011 ▸) with *XDS* (Kabsch, 2010 ▸) were chosen for refinement. Data reduction and scaling were carried out with *AIMLESS* (Evans & Murshudov, 2013 ▸).

Initial phases were assigned by molecular replacement using *Phaser* (McCoy *et al.*, 2007 ▸). PDB entries 2ili and 3dcc were used as starting models for CA II and the CA IX mimic, respectively (Fisher *et al.*, 2007 ▸; Genis *et al.*, 2009 ▸). Ligand dictionaries and restraints were generated using *ELBOW* (Moriarty *et al.*, 2009 ▸) and model building was performed using *Coot* (v.0.8.9.1; Emsley *et al.*, 2010 ▸) with rounds of iterative refinement using the *phenix.refine* GUI (v.1.18.2-3874; Afonine *et al.*, 2012 ▸). *mF*_o_ − *DF*_c_ difference maps were calculated in *Phenix* (Afonine *et al.*, 2012 ▸) and visually inspected in *Coot* (Emsley *et al.*, 2010 ▸). For water placement a 0.7σ cutoff was used to capture weakly bound waters, but they were only retained in the final model if there was no residual negative electron density after refinement. The final models and the corresponding structure factors were deposited in the PDB and the details can be found in Table 1 ▸.

### 2.5. AZM and SAHA binding interactions and effects on thermal stability

WT CA II and the CA IX mimic were diluted to a concentration of ∼1 mg ml^−1^ in 50 m*M* Tris–HCl pH 8.0. SAHA was dissolved in pure DMSO to the following concentrations: 100 m*M*, 10 m*M*, 1 m*M*, 100 µ*M* and 10 µ*M*. These SAHA stocks were then further tenfold diluted by adding 1.5 µl of the series to 13.5 µl protein solution, resulting in 10% DMSO in the final solution. Acetazolamide was prepared in the same way and run as a control inhibitor of CA. After ∼1 h incubation at room temperature, the samples were placed in standard capillaries and loaded into a Prometheus NT.48 (NanoTemper Technologies GmbH, Germany). The instrument reads changes in the intrinsic tryptophan and tyrosine fluorescence (350 nm/330 nm fluorescence ratio) as an indicator of protein unfolding during heating. The samples were heated at a rate of 1°C min^−1^ from 20 to 95°C (293 to 368 K). The melting temperatures (*T*_m_) were calculated using the on-board *PR.ThermControl* software. The melting temperatures were determined by automatically fitting the measured curves to a polynomial function, where the slope maximum is shown as a peak of its first derivative.

To calculate the interactions, buried surface area and associated Δ*G* (kcal mol^−1^) between AZM and SAHA in the CA active sites and zinc, the structures from this work were uploaded to the *Arpeggio* (https://biosig.lab.uq.edu.au/arpeggioweb/) and *PDBePISA* (https://www.ebi.ac.uk/pdbe/pisa/) servers (Krissinel & Henrick, 2007 ▸; Jubb *et al.*, 2017 ▸). The data are shown in Tables 2 ▸ and 3 ▸.

## 3. Results and discussion

### 3.1. SwissDOCK

**Figure 2:**
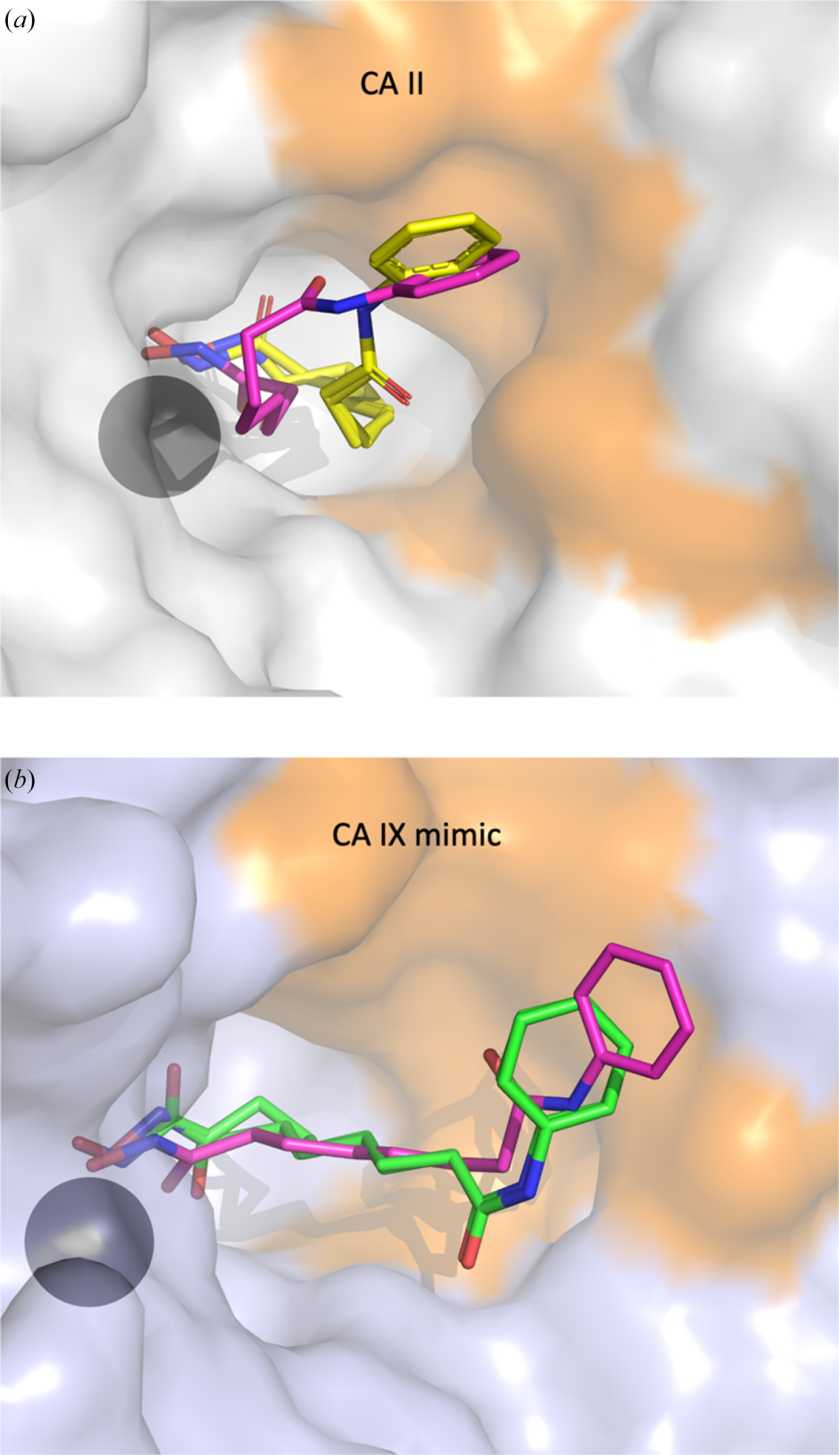
*SwissDock*-predicted SAHA binding mode to CA compared with the crystal structure. (*a*) CA II is shown in gray surface representation with the SAHA molecule shown in yellow (crystal structure) and magenta (modeling) ball-and-stick representation. (*b*) The CA IX mimic is shown in blue surface representation with the SAHA molecule shown in green (crystal structure) and magenta (*SwissDOCK* modeling) ball-and-stick representation. The hydrophobic area around the SAHA alkyl chain and benzene ring is shaded orange in the surface representation. This figure was generated using *PyMOL* v.2.5.2 (Schrödinger).

To assess the feasibility of preparing complexes between CA and SAHA, docking was performed with *SwissDOCK* using the *With Attracting Cavities* module (Röhrig *et al.*, 2023 ▸; Bugnon *et al.*, 2024 ▸). For both the WT and mimic models, *SwissDOCK* produced reasonable binding solutions with the hydroxamate moiety of SAHA pentacoordinated to the zinc and the body of the molecule readily fitting in the wide, conical CA active site (Fig. 2 ▸). While the predicted zinc coordination was similar to the experimental complexes of HDAC and SAHA (PDB entry 4lxz; Lauffer *et al.*, 2013 ▸), there were some deviations in the predicted hexyl linker and phenylamide locations compared with the experimentally determined crystal structures for CA. The predicted and experimentally determined positions for SAHA in CA II and the CA IX mimic are shown as overlays in Fig. 2 ▸.

### 3.2. Crystal preparation of SAHA with CA II and the CA IX mimic

The first attempts to simply soak the crystals with a 1:10 molar ratio of protein to SAHA failed and crystals were marred due to the presence of 10% DMSO in the drops. Co-crystallization by pre-mixing protein and SAHA in a 1:10 molar ratio, incubating for 1 h, centrifuging any precipitate and then using the supernatant for subsequent crystallization-drop preparation only produced crystals for CA II. To avoid the presence of DMSO, 2 µl aliquots of 100 m*M* SAHA dissolved in 100% DMSO were dispensed into sitting-drop plates (Linbro 24-well) and left to evaporate in a fume hood overnight. The next day, 20 µl crystallization drops including crystals were moved onto the dried SAHA and left for a week prior to data collection.

### 3.3. Crystallographic data

Diffraction data were collected for CA–SAHA complexes prepared in different ways as described above. Data-collection and refinement statistics are shown in Table 1 ▸. WT-T1 and Mimic-T1 correspond to crystals obtained after transferring the entire apo drop to dried SAHA in sitting-drop wells for WT CA II and the CA IX mimic, respectively. Furthermore, crystals were obtained from co-crystallization setups for WT only (WT-CC1). The WT-T1, WT-CC1 and Mimic-T1 data sets were collected at room temperature. All crystals diffracted well, with the data extending to ∼1.4 Å resolution in all cases. To avoid excessive radiation damage, data collection was limited to 1800 images for the room-temperature experiments. As there was some disorder in part of the bound SAHA in the room-temperature Mimic-T1 structure, the experiment was repeated at 100 K to ‘freeze in’ an orientation of the benzene ring (Mimic-T3). The data-collection temperature did not make a difference, and the same relative disorder was also observed at 100 K. Since the structures are highly homologous and the diffraction data extended to similar resolution, all structures were refined to 1.4 Å resolution to enable side-by-side comparison. The binding conformation of SAHA was nearly identical between data sets of CA II or the CA IX mimic. Therefore, to simplify the representation and comparison of the WT and mimic complexes, only the WT-T1 and Mimic-T1 structures were used as representatives in the figures in this manuscript; however, all four structures were deposited in the PDB with the codes shown in Table 1 ▸. Globally, both structures were experimentally identical to previously determined structures of CA II (r.m.s.d. of 0.21 Å compared with PDB entry 2ili) and the CA IX mimic (r.m.s.d. of 0.12 Å compared with PDB entry 3dcc).

### 3.4. SAHA binding conformers

**Figure 3:**
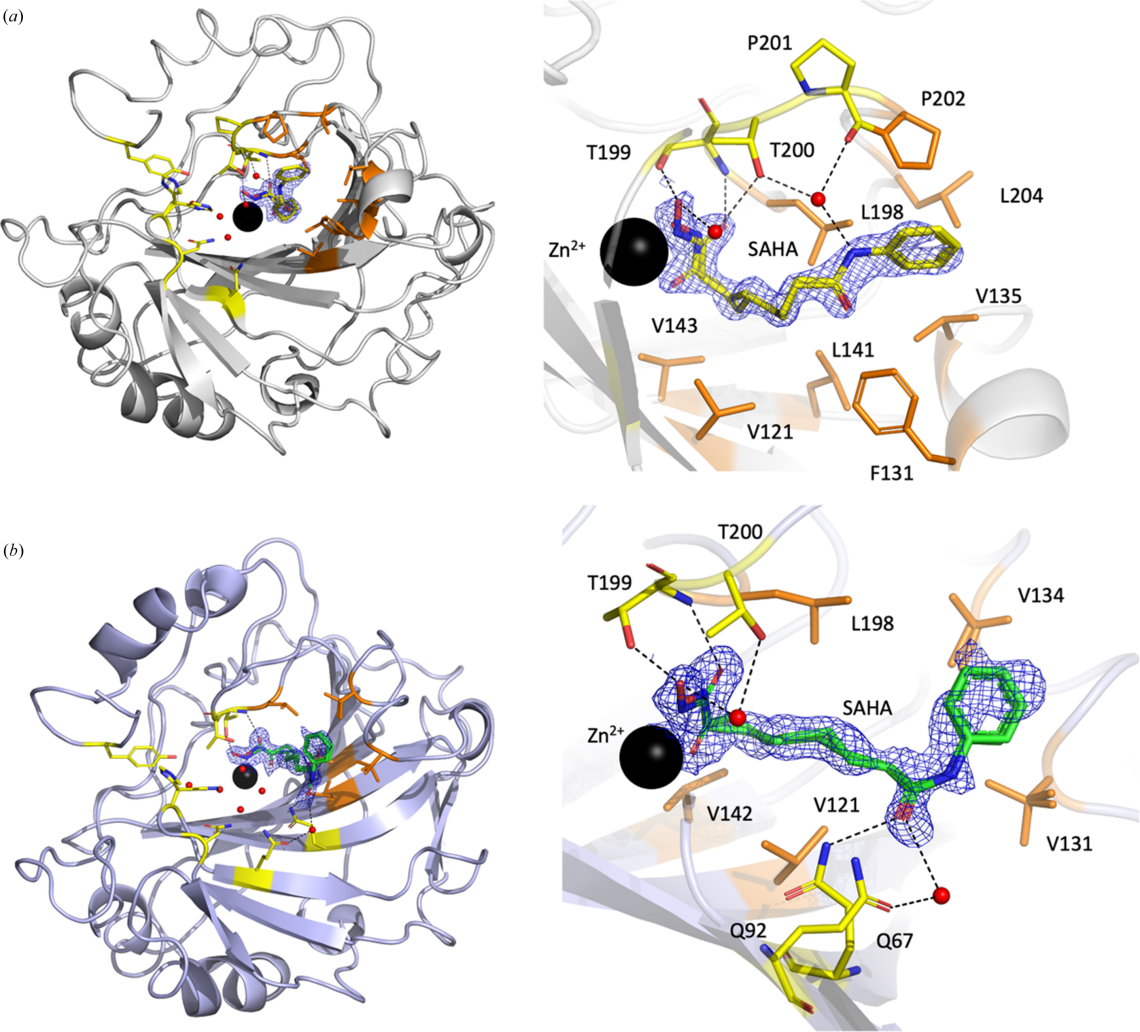
Binding interactions between CA and SAHA. (*a*) The CA II structure is shown as a gray cartoon; the active-site Zn^2+^ and water molecules are shown as black and red spheres, respectively. Active-site residues and SAHA are shown in yellow ball-and-stick representation. (*b*) The CA IX mimic structure is shown as a blue cartoon; the active-site Zn^2+^ and water molecules are shown as black and red spheres, respectively. Active-site residues and SAHA are shown in yellow and green ball-and-stick representation, respectively. Hydrophobic residues around the SAHA alkyl chain and benzene ring are shown in orange ball-and-stick representation. 2*mF*_o_ − *DF*_c_ electron density for SAHA is shown as a blue mesh and is contoured to 1.0σ in both panels. Hydrogen bonds are indicated by black dashed lines. Zinc ligands (His94, His96 and His119) and His64 are omitted for clarity. This figure was generated using *PyMOL* v.2.5.2 (Schrödinger).

In both CA II structures, dual conformers with an approximately 50/50 split occupancy of SAHA in two different zinc-coordination modes support the experimental data best (Fig. 3 ▸*a*), while for both CA IX mimic structures dual conformers with an approximately 60/40 split occupancy of SAHA in two different zinc-coordination modes support the experimental data best (Fig. 3 ▸*b*). In both the A and B conformations, the hydroxamate group displaces the zinc-bound water (ZW) and deep water (DW), while the rest of the hydrogen-bonded water network (W1, W2, W3a and W3b), essential for proton transfer, remains intact in the hydrophilic part of the active site. The hexyl linker and phenylamide moieties of SAHA bind in the hydrophobic part of the CA II active site.

### 3.5. Specific interaction between SAHA and WT CA II

SAHA conformer A binds with a pentahedral zinc coordination where both O1 and O2 bind to the zinc (Fig. 3 ▸*a*). For the A conformer it is the zinc interaction that dominates the binding interaction, with a possible hydrogen bond between O1 and the Thr199 hydroxyl group (∼2.8 Å). The hydrophobic hexyl linker and terminal benzene ring are accommodated in the hydrophobic part of the active site (Supplementary Fig. S4*a*). This area is composed of residues Val121, Phe131, Leu141 and Val143. In both of the CA II structures (WT-T1 and WT-CC1) the phenylamide group of SAHA makes a water-mediated hydrogen bond to the Pro202 carbonyl and the hydroxyl group of Thr200.

Conformer B binds with N1 in a tetrahedral zinc coordination with a more complicated network of putative hydrogen bonds. O2 makes a bifurcated hydrogen bond to the hydroxyl group of Thr199 and a water molecule. O1 of the B conformer makes a hydrogen bond to the amide backbone of Thr199. The rest of the molecule binds in the same conformation as A where the phenylamide water-mediated hydrogen bond to the Pro202 carbonyl and the hydroxyl group of Thr200 is maintained.

For both the A and B SAHA conformers bound to CA II, the terminal benzene ring is accommodated in a hydrophobic region composed of Phe131, Val135, Pro202 and Leu204 (Supplementary Fig. S4*a*). Compared with the mimic structure discussed below, the binding conformation of SAHA is reasonably well defined in the wild-type CA II structure. Supplementary Fig. S2(*a*) shows an *mF*_o_ − *DF*_c_ omit map, while Supplementary Fig. S3(*a*) shows residual positive and negative *mF*_o_ − *DF*_c_ electron density for SAHA in CA II contoured at ±3σ. These maps indicate that the refined position of SAHA fits the experimental data well.

### 3.6. Specific interaction between SAHA and the CA IX mimic

**Figure 4:**
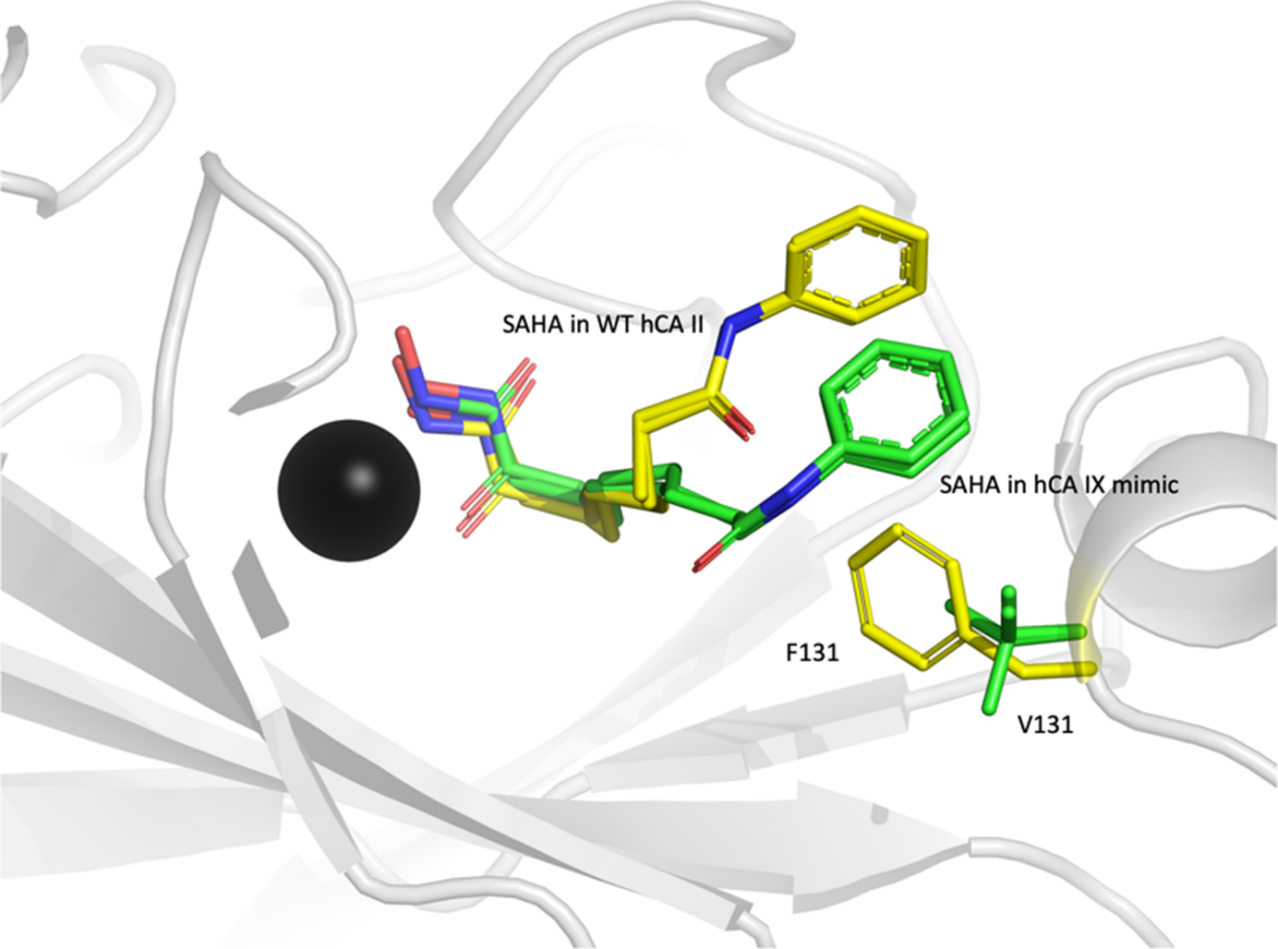
Overlay of the SAHA binding mode in CA II and the CA IX mimic. For reference, CA II is shown as a gray cartoon with the Zn^2+^ ion as a black sphere. SAHA and the hydrophobic residue 131 (Phe in CA II and Val in the CA IX mimic) for CA II and the CA IX mimic are shown in yellow and green ball-and-stick representation, respectively. This figure was generated using *PyMOL* v.2.5.2 (Schrödinger).

Similar to both WT complexes, the CA IX mimic structure shows that SAHA binds in two overlapping conformers with two different zinc-coordination modes: pentahedral and tetrahedral. The interactions and hydrogen bonds are conserved between the WT and the CA IX mimic up until the benzene ring (Fig. 3 ▸*b*). The amino-acid residues are different between the two proteins in this region; specifically, Phe131 in WT is Val131 in the CA IX mimic (Fig. 4 ▸; Supplementary Figs. S4*b* and S4*c*). The absence of the bulky hydrophobic Phe side chain leads to the benzene ring being more disordered and refined to an orientation with high *B* factors and weak 2*mF*_o_ − *DF*_c_ density (Fig. 3 ▸). This disorder can also be seen in *mF*_o_ − *DF*_c_ omit density maps (Supplementary Fig. S2*b*), where there is no density at the 3σ level for the benzene ring and some residual negative *mF*_o_ − *DF*_c_ density after refinement (Supplementary Fig. S3*b*). The final conformation of SAHA in the deposited structure was the result of many rounds of refinement and attempts to place it within acceptable geometric constraints. Since the initial structure was determined at room temperature and this may contribute to the relative disorder of the benzene moiety, we repeated the structure determination at cryogenic temperatures. However, even at 100 K the structure was highly homologous to the room-temperature structure and the terminal region of SAHA was still relatively disordered. Therefore, the temperature of data collection made no difference with regard to the conformation of the phenylamide group.

### 3.7. SAHA binding buried surface area and melting temperature (*T*_m_)

The results from the *PDBePISA* server show that the buried surface area between SAHA and CA II and the CA IX mimic are comparable at ∼290 and 297 Å^2^, respectively (Table 2 ▸; Krissinel & Henrick, 2007 ▸). To verify that there are no differences in the zinc-coordination buried surface area for SAHA binding, these values were compared for CA and HDAC structures and are comparable between all structures at ∼30 Å^2^ (Table 2 ▸). Interestingly, when comparing the SAHA binding to CA to that of the target HDAC7/8 the energetics are more favorable for binding to CA. While the buried surface area between SAHA and the target proteins are comparable between CAs and HDACs, the differences in the calculated Δ*G* are large, especially since Δ*G* is logarithmically related to the equilibrium dissociation constant (*K*_d_). These predications would suggest that SAHA has a higher binding affinity to CAs than HDACs, its intended target. Intermolecular interactions between CA and either AZM or SAHA were calculated using the *Arpeggio* server (Jubb *et al.*, 2017 ▸). The results are summarized in Table 3 ▸. As expected, the larger SAHA molecule with the hexyl linker and phenylamide group makes more extensive hydrophobic interactions compared with AZM. The calculations also illustrate what we observe in the CA II and CA IX structures: the substitution at position 131 influenced the position and interactions between the phenylamide group of SAHA and CA.

**Figure 5:**
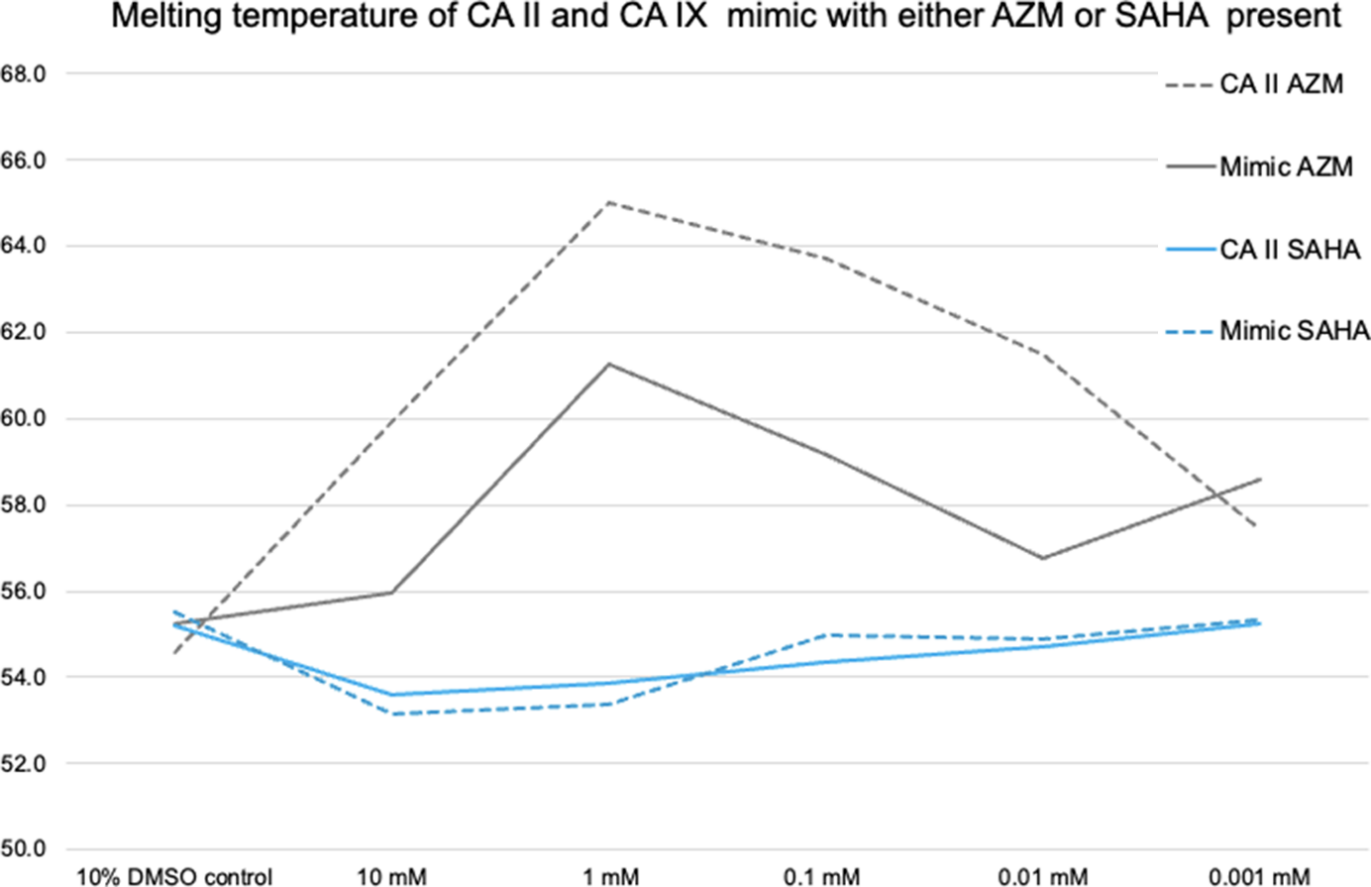
Melting temperatures as measured by nanoDSF for CA II and the CA IX mimic in the presence of either acetazolamide (AZM) or SAHA. The data are also shown in a tabular format in Supplementary Table S1.

To assess whether SAHA binding, as observed in the crystal structures, had a stabilizing or destabilizing effect on CA, we performed a series of nanoDSF measurements with different SAHA and matching AZM concentrations. We included AZM as a control since AZM is a well characterized nanomolar inhibitor of CA. The results for both WT CA II and the CA IX mimic can be seen in Fig. 5 ▸ and Supplementary Table S1. After controlling for the presence of DMSO, no significant effect of SAHA on CA II or the CA IX mimic could be seen. There is perhaps a slight destabilization of a few degrees at the highest SAHA concentrations (1–10 m*M*). This is in contrast to AZM, which has a ∼10°C stabilization effect on CA II and about 6°C on the CA IX mimic. This would suggest that SAHA is not as tight-binding as AZM or reflects the different set of interactions that mediate SAHA binding compared with AZM (Fig. 5 ▸, Supplementary Table S1). However, without additional measurements of the affinity of inhibition, further conclusions cannot be made based on the nanoDSF data alone. The limitations of this method are discussed in Coyle & Walser (2020 ▸).

## 4. Conclusions

In this work, we report crystal structures of a clinically used HDACi, SAHA, bound to the previously unidentified off-target proteins CA II and CA IX. Prior to crystallization and soaking trials, modeling using *SwissDOCK* indicated that HDACi could bind to the CA active sites. *SwissDOCK* was also able to correctly predict the pentahedral coordination of the hydroxamate moiety to the active zinc, as later observed in the crystal structures. Comparison of the CA II and the CA IX mimic active-site modeling/docking results show that the predicted binding took a mutation into account by placing the SAHA benzene ring towards the area vacated by the Phe131-to-Val substitution. Close inspection of the binding of SAHA reveals that there are two overlapping binding configurations around the zinc and that there are differences in the location of the hexyl linker tail and the location of phenylamide group between CA II and the CA IX mimic. The observed dual zinc-coordinating modes of SAHA to CA are not present in the HDAC crystal structure (PDB entry 4lxz; Lauffer *et al.*, 2013 ▸). This is probably driven by a complex electrostatic environment in that the zinc-coordination sites are quite different between HDAC and CA. The former uses two Asp residues and a single His residue to bind the zinc, while CA uses three His residues. Beyond the primary zinc-coordinating residues, there is also quite some variability in how solvent-exposed the active sites are. For example, in HDAC (PDB entry 4lxz) there is a narrow occlusion where two Phe residues sandwich part of the alkyl chain. This is in contrast to the area adjacent to the alkyl chain in CA where there is ordered solvent leading to the bulk solvent outside the active site. Interestingly, in both the cryo and room-temperature CA IX mimic crystal structures the phenylamide group was equally disordered. Since there is a Val residue in the mimic instead of the bulky, hydrophobic Phe found in the WT, this is interpreted as rotational freedom of the ligand due to the lack of any steric hindrance from the protein.

It is interesting that free-energy calculations support the experimental observation that SAHA binds to CA perhaps as well as to the target HDAC. However, since these calculations were predicted from the structure of CA soaked with SAHA at high concentration for an extended period of time, it is possible that in solution the binding affinity is weaker than predicted. Due to our experimental design, we cannot exclude the possibility that the conformation that we captured in our crystal structure has a very slow on-rate or a fast off-rate in solution.

SAHA binds to CAs in a similar way as other CAi, such as AZM, by displacing the catalytic zinc-bound solvent; thus, it is reasonable to expect the binding interaction to be inhibitory to the CA enzymatic activity. Unfortunately, due to the low solubility of SAHA in aqueous solutions, we were unable to measure a binding affinity using isothermal titration calorimetry. Furthermore, since hydroxamate ligands react with 4-nitrophenyl acetate, the ester reporter substrate commonly used to measure CA activity, we were unable to measure an inhibition constant (Ghosh *et al.*, 2005 ▸). In future experiments, it would be interesting to see whether SAHA changes the intracellular pH of cells, similar to SLC-0111, the CAi currently in clinical trials. If so, the effect of SAHA on hypoxic tumors could perhaps be evaluated.

Clinically, HDACi are administered orally and intra­venously and would be able to bind to CAs, which are abundantly spread throughout all organs and tissues in the body. This could contribute to the side effects of SAHA through dysregulation of the physiological pH. Furthermore, off-target binding to CAs could also reduce the amount of free SAHA available for HDAC inhibition. It would be interesting to determine whether next-generation HDACi also exhibit off-target binding to CAs due to the similar active site between HDACs and CAs.

Finally, the hydroxamate moiety presents an alternative, nonsulfonamide anchor for designing CAi. However, the binding of SAHA to both CA II and the CA IX mimic highlights the challenges of designing isoform-selective drugs against highly conserved CAs.

## 5. Related literature

The following references are cited in the supporting information for this article: Madeira *et al.* (2024 ▸) and Robert & Gouet (2014 ▸).

## Supplementary Material

PDB reference: carbonic anhydrase IX mimic in complex with vorinostat, 9o8x

PDB reference: 9oaf

PDB reference: carbonic anhydrase II in complex with vorinostat, co-crystal, 9oam

PDB reference: drug soak, 9obj

Supplementary Table and Figures. DOI: 10.1107/S2053230X25007447/dp5133sup1.pdf

## Figures and Tables

**Table 1:** Crystallographic data-collection and refinement statistics Values in parentheses are for the highest resolution shell.

	WT-co-crystal (WT-CC1)	WT-transfer (WT-T1)	Mimic-transfer (M-T1)	Mimic-transfer (M-T3)
PDB code	9oam	9obj	9oaf	9o8x
Data collection				
Temperature (K)	293	293	293	100
Wavelength (Å), transmission (%)	0.7293, 20	0.7293, 20	0.7293, 20	0.7293, 40
No. of images, oscillation (°)	1800, 0.1	1800, 0.1	1800, 0.1	3600, 0.1
Space group	*P*2_1_	*P*2_1_	*P*2_1_	*P*2_1_
*a*, *b*, *c* (Å)	42.91, 41.75, 72.78	42.92, 41.77, 72.88	42.6, 41.67, 72.58	42.25, 41.33, 71.80
α, β, γ (°)	90.0, 104.5, 90.0	90.0, 104.5, 90.0	90.0, 104.0, 90.0	90.0, 103.9, 90.0
Resolution range (Å)	24.9–1.40 (1.45–1.40)	25.6–1.40 (1.45–1.40)	24.7–1.40 (1.45–1.40)	26.64–1.40 (1.40–1.45)
No. of reflections	164796 (12132)	165250 (12093)	156652 (11411)	321192 (31544)
No. of unique reflections	48426 (4152)	48418 (4199)	47019 (3712)	47090 (4632)
Multiplicity	3.4 (2.9)	3.4 (2.9)	3.3 (3.1)	6.8 (6.8)
Mean *I*/σ(*I*)	10.0 (3.2)	11.5 (3.4)	10.4 (2.5)	16.9 (3.1)
Completeness (%)	98.1 (84.8)	97.9 (85.9)	96.2 (76.9)	98.9 (98.1)
*R*_merge_ (all)	0.076 (0.336)	0.069 (0.411)	0.065 (0.572)	0.068 (0.669)
CC_1/2_	0.994 (0.832)	0.995 (0.770)	0.996 (0.687)	0.999 (0.725)
Refinement				
*R*_cryst_/*R*_free_	0.152/0.171	0.152/0.176	0.154/0.174	0.157/0.179
No. of atoms: protein, solvent, zinc, ligand	2085, 230, 1, 38	2137, 242, 1, 38	2149, 223, 1, 38	2129, 366, 1, 38
Ramachandran favored, allowed, outliers (%)	97, 3, 0	97, 3, 0	98, 2, 0	97, 3, 0
Rotamer outliers (%)	0.5	0.9	0.9	0.9
Average *B* factor: protein, solvent, zinc, ligand (Å^2^)	19.16, 35.93, 9.02, 29.50	18.30, 35.39, 8.81, 29.31	22.03, 38.21, 10.51, 43.31	17.70, 33.07, 9.73, 29.53
R.m.s.d. bond lengths (Å), angles (°)	0.006, 0.924	0.007, 0.958	0.012, 1.309	0.015, 1.593
Clashscore	0	1	2	3
Real-space correlation	0.942	0.945	0.941	0.934

**Table 2:** *PDBePISA* calculations of AZM and SAHA binding energy and buried surface area upon binding to the protein target (Krissinel & Henrick, 2007 ▸)

PDB code	Protein (ligand)	Binding interface area (Å^2^)	Δ*G* (kcal mol^−1^)	Zinc binding interface area (Å^2^)	Zinc Δ*G* (kcal mol^−1^)
4lxz †	HDAC2 (SAHA)	289.8	1.7	31.2	−13.1
3c0z ‡	HDAC7 (SAHA)	190.8	2.3	31.3	−13.0
1t69	HDAC8 (SAHA)	249.4	3.6	30.6	−13.8
This work	CA II (SAHA)	300.3	−3.6	30.7	−13.2
This work	CA IX mimic (SAHA)	297.4	−4.1	30.8	−13.0
3hs4	CA II (AZM)	209.5	1.3	29.1	−11.6
3dc3	CA IX mimic (AZM)	224.7	−1.0	31.5	−12.8

† Average of three bound SAHA molecules to three protein chains.

‡ The phenylamide moiety of SAHA was not modeled in the PDB entry.

**Table 3:** Summary of calculated molecular interactions between AZM and SAHA and CA using the *Arpeggio* server (Jubb *et al.*, 2017 ▸)

	CA II + SAHA	CA IX mimic + SAHA	CA II + AZM	CA IX mimic + AZM
Total No. of contacts	240	233	222	180
Hydrogen bonds	3	5	4	4
Water-mediated hydrogen bonds	11	11	7	4
Hydrophobic contacts	28	17	7	4
