# Supplementary material for: Off-target binding of the histone deacetylase inhibitor vorinostat to carbonic anhydrase II and IX
Source: Acta Crystallogr F Struct Biol Commun. 2025 Aug 26;81(Pt 9):388–97. doi: 10.1107/S2053230X25007447 (PMC12400194; doi:10.1107/S2053230X25007447)
Supplement: Supplementary file 1 [file f-81-00388-sup1.pdf]

STRUCTURAL BIOLOGY COMMUNICATIONS

**Volume 81 (2025)**

**Supporting information for article:**

# Off-target binding of the histone deacetylase inhibitor vorinostat to carbonic anhydrase II and IX

**Mitchell C. Gulkis, James T. Hodgkinson, Céleste P. Sele, Wolfgang Knecht, Robert McKenna and S. Zoë Fisher**

**Table S1** Melting temperatures of CA II and CA IX mimic using nanoDSF and a concentration series of AZM and SAHA

AZM and SAHA were prepared in 100% DMSO with 10% DMSO present in final samples used for nanoDSF. Measurements were done in triplicate and average Tm with standard deviation for each compound is shown in parentheses.

| | **AZM or SAHA concentration** | **AZM Tm (°C)** | **SAHA Tm (°C)** |
|---|---|---|---|
| **CA II** | 0 mM (10% DMSO control) | 54.6 (0.32) | 55.2 (0.81) |
| | 0.001 mM | 57.5 (0.10) | 55.3 (0.30) |
| | 0.01 mM | 61.5 (0.15) | 54.7 (0.17) |
| | 0.1 mM | 63.7 (0.06) | 54.3 (0.16) |
| | 1 mM | 65.0 (0.20) | 53.9 (0.18) |
| | 10 mM | 59.9 (0.77) | 53.6 (0.23) |
| | | | |
| **CA IX mimic** | 0 mM (10% DMSO control) | 55.2 (0.06) | 55.5 (0.41) |
| | 0.001 mM | 58.6 (0.04) | 55.3 (0.13) |
| | 0.01 mM | 56.7 (0.05) | 54.9 (0.23) |
| | 0.1 mM | 59.2 (0.19) | 55.0 (0.25) |
| | 1 mM | 61.3 (0.27) | 53.4 (0.02) |
| | 10 mM | 56.0 (0.78) | 53.2 (0.14) |

>CAII

MSHHWGYGKH NGPEHWHKDF PIAKGERQSP VDIDTHTAKY DPSLKPLSVS
YDQATSLRIL NNGHAFNVEF DDSQDKAVLK GGPLDGTYRL IQFHFHWGSL
DGQGSEHTVD KKKYAAELHL VHWNTKYGDF GKAVQQPDGL AVLGIFLKVG
SAKPGLQKVV DVLDSIKTKG KSADFTNFDP RGLLPESLDY WTYPGSLTTP
PLLECVTWIV LKEPISVSSE QVLKFRKLNF NGEGEPEELM
VDNWRPAQPL KNRQIKASFK

>CAIX-mimic

MSHHWGYGKH NGPEHWHKDF PIAKGERQSP VDIDTHTAKY DPSLKPLSVS
YDQATSLRIL NNGHSFQVTF DDSQDKAVLK GGPLDGTYRL LQFHFHWGSL
DGQGSEHTVD KKKYAAELHL VHWNTKYGDV GKAVQQPDGL AVLGIFLKVG
SAKPGLQKVV DVLDSIKTEG KSADFTNFDP RGLLPESLDY WTYPGSLTTP
PLAECVTWIV LKEPISVSSE QVLKFRKLNF NGEGEPEELM VDNWRPAQPL
KNRQIKASFK

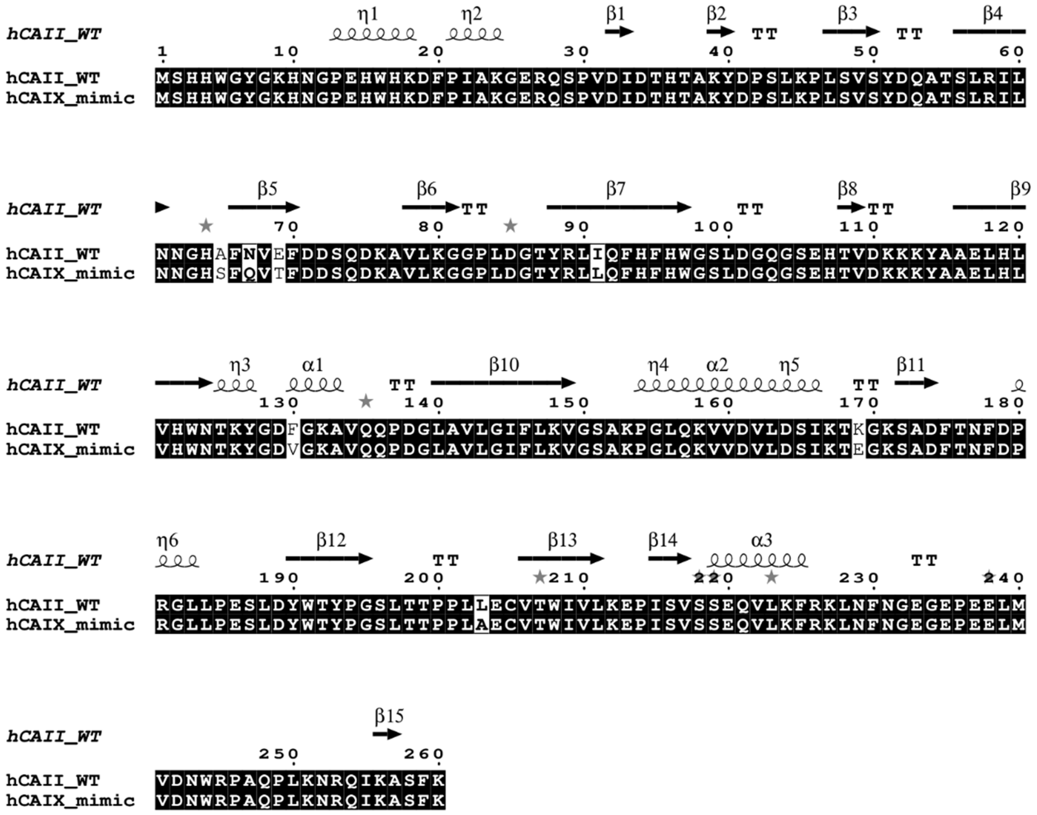


**Figure S1** FASTA sequences of CA II and CA IX mimic. The residues that are changed in the CA active site to match CA IX are highlighted in yellow in both sequences. Alignment was generated using Clustal Omega and displayed in ESPript [Madeira et al., 2024; Robert et al., 2014]. The substitutions in WT to generate the mimic are: A65S, N67Q, E69T, I91L, F131V, K170E, L204A.

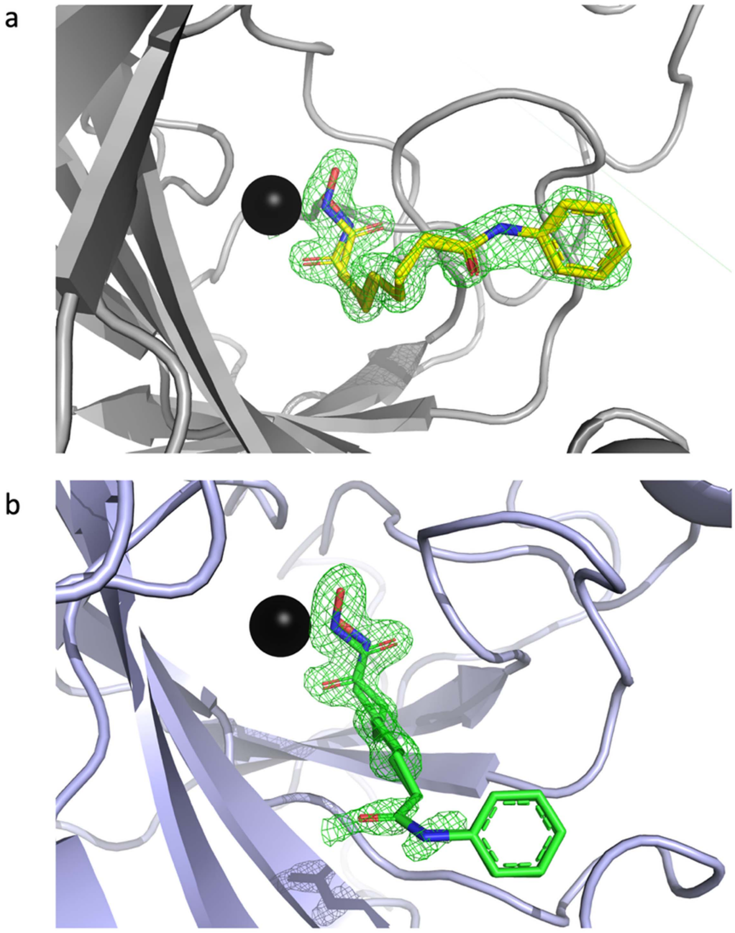


**Figure S2** Presence of SAHA in CA II and CA IX mimic. (a) The CA II structure is shown in gray cartoon with the active site zinc ion as a black sphere. SAHA is shown in yellow ball-and-stick representation. (b) The CA IX mimic structure is shown in blue cartoon, with the active site zinc as a black sphere. SAHA is shown in green ball-and-stick. $mF_o$-$DF_c$ omit electron density for SAHA is shown in green mesh and is contoured to 3.0 sigma in both panels.

a

b

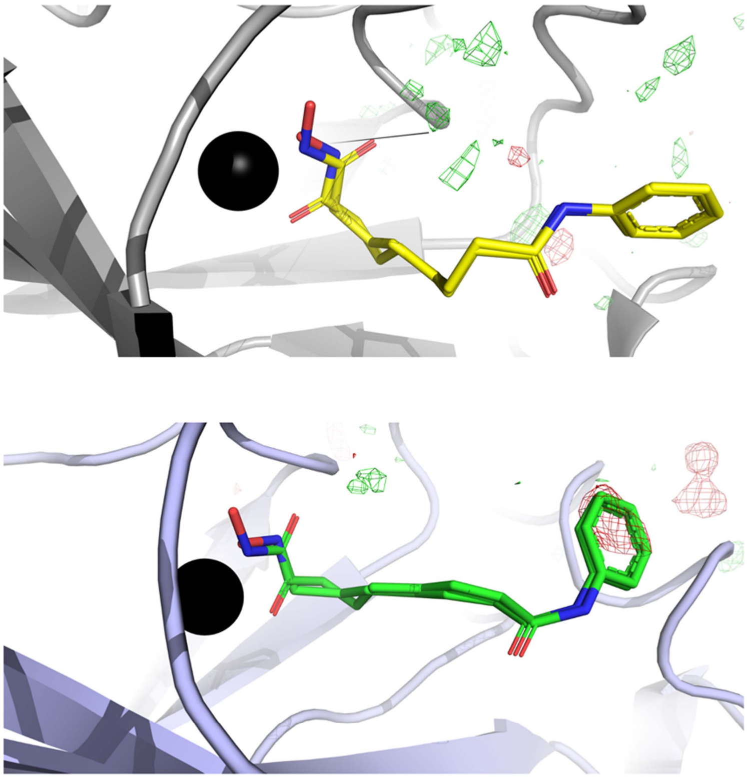

**Figure S3** Presence of SAHA in CA II and CA IX mimic. (a) The CA II structure is shown in gray cartoon with the active site zinc ion as a black sphere. SAHA is shown in yellow ball-and-stick representation, (b) The CA IX mimic structure is shown in blue cartoon, with the active site zinc as a black sphere. SAHA is shown in green ball-and-stick. Residual positive (green) and negative (red) $mF_o$-$DF_c$ electron density after refinement for SAHA is shown in mesh representation and is contoured to +/- 3 sigma.

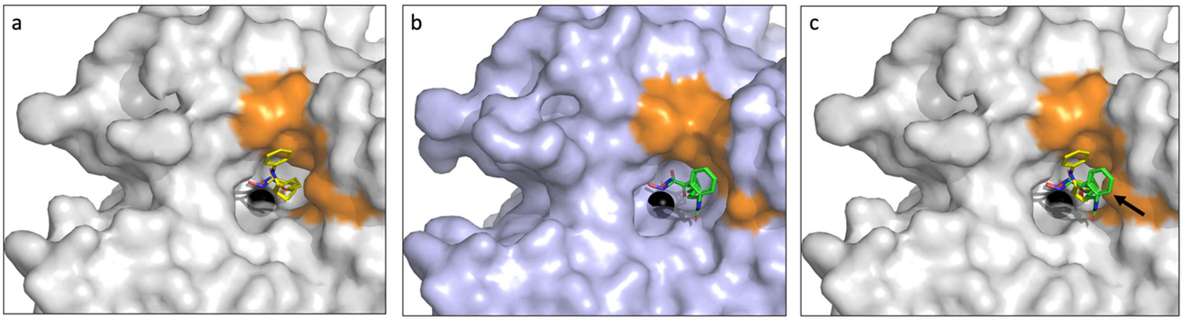


**Figure S4** CA in complex with SAHA. a) CA II is shown as a gray surface with the hydrophobic part of the active site shaded in orange (Val121, Phe131, Val135, Leu141, Ala142, Leu198,Pro202, and Leu204) with SAHA in yellow ball-and-stick representation; b) CA IX mimic is shown in blue surface with the hydrophobic part of the active site shaded in orange (Val121, Val131, Val135, Leu141, Ala142, Leu198, Pro202, Ala204) with SAHA in green ball-and-stick representation; c) overlay of the SAHA binding modes onto CA II shows the steric clash (black arrow) between the benzene ring and bump of the phenyl ring caused by the side chain of Phe131. Figure was generated using PyMol v. 2.5.2 [Schrödinger LLC].
